# Deep phenotyping for precision medicine in Parkinson's disease

**DOI:** 10.1242/dmm.049376

**Published:** 2022-06-01

**Authors:** Ann-Kathrin Schalkamp, Nabila Rahman, Jimena Monzón-Sandoval, Cynthia Sandor

**Affiliations:** UK Dementia Research Institute at Cardiff University, Division of Psychological Medicine and Clinical Neuroscience, Haydn Ellis Building, Maindy Road, Cardiff CF24 4HQ, UK

**Keywords:** Genetics, Phenotyping, Precision medicine

## Abstract

A major challenge in medical genomics is to understand why individuals with the same disorder have different clinical symptoms and why those who carry the same mutation may be affected by different disorders. In every complex disorder, identifying the contribution of different genetic and non-genetic risk factors is a key obstacle to understanding disease mechanisms. Genetic studies rely on precise phenotypes and are unable to uncover the genetic contributions to a disorder when phenotypes are imprecise. To address this challenge, deeply phenotyped cohorts have been developed for which detailed, fine-grained data have been collected. These cohorts help us to investigate the underlying biological pathways and risk factors to identify treatment targets, and thus to advance precision medicine. The neurodegenerative disorder Parkinson's disease has a diverse phenotypical presentation and modest heritability, and its underlying disease mechanisms are still being debated. As such, considerable efforts have been made to develop deeply phenotyped cohorts for this disorder. Here, we focus on Parkinson's disease and explore how deep phenotyping can help address the challenges raised by genetic and phenotypic heterogeneity. We also discuss recent methods for data collection and computation, as well as methodological challenges that have to be overcome.

## Abstract (precis)

**Summary:** This Review discusses challenges in neurodegenerative disease research and how deep phenotyping and genomics, alongside machine learning, can help the progress towards precision medicine.

## Introduction

To elucidate the genetic and molecular processes that contribute to disease, the research community has made considerable efforts to develop large case/control genetic studies. These have been remarkably successful in identifying common genetic risk variants associated with various disorders. Over 6000 genome-wide association studies (GWAS; see Glossary, Box 1) have been published for over 1000 traits that report on tens of thousands of genetic risk variants (Watanabe et al., 2019; https://www.ebi.ac.uk/gwas/). However, they do not fully explain why some carriers of risk alleles do not develop the associated disorder and why people who carry similar risk alleles develop distinct phenotypes.

A critical challenge in medicine is to understand why patients diagnosed with the same disorder vary in their clinical presentation. This is especially true for Parkinson's disease (PD), for which the age of onset, rate of progression, and type and severity of symptoms differ among the 9.3 million people worldwide who live with this disorder (Maserejian et al., 2020). As the frequency of the misdiagnosis of PD is particularly high, ∼30% (Beach and Adler, 2018), and its consequences are dramatic, it is crucial to identify the aetiology of this clinical heterogeneity. One of the challenges of studying PD is that direct access to the relevant tissue, the brain, is limited. In addition, a long prodromal phase (Box 1) precedes the first clinical symptoms, and 90% of cases are considered sporadic, with an assumed genetic heritability of ∼30% (Keller et al., 2012).

**Box 1. Glossary**

**Apathy:** a lack of motivation.

**Bradykinesia:** a slowness of movement, one of the clinical hallmark symptoms of Parkinson's disease.

**Classification:** a group of supervised machine learning methods that predict a discrete outcome (category) based on a set of variables.

**Clustering:** a group of unsupervised machine learning methods that groups data points into clusters. Objects in the same cluster are similar to one another and less similar to objects from other clusters.

**Convolutional neural network (CNN):** a type of neural network often used to analyse visual imagery where neighbouring inputs are considered together.

**Deep learning (DL):** a subdivision of machine learning in which neural networks, which are computational methods inspired by biological neural networks, with multiple layers extract increasingly high-level features from raw input.

**Dimensionality reduction:** a group of unsupervised machine learning methods that transform high-dimensional data into a low-dimensional representation that retains most of the information.

**Diplopia:** simultaneous perception of two images from one object.

**Dosage effect:** Change in a phenotype due to alternations in the dose/amount of the product of a gene.

**Dyskinesia:** involuntary, uncontrolled muscle movements.

**Dysphagia:** difficulty swallowing.

**Genome-wide association studies (GWAS):** statistical, hypothesis-free methods to test for the association of genetic loci and phenotypic traits.

**Hyposmia:** reduced ability to smell.

**Latent class:** a group of unsupervised machine learning methods that relate observations to latent factors that are assumed to cause the observations.

**Mendelian randomisation:** a method to test for putative causal relationships between modifiable risk factors and diseases.

**Molecular neuroimaging:** techniques to visualise molecular or cellular processes in the brain through a probe or imaging agent that creates a signal through the interaction with the event of interest.

**Neuronopathy:** a subgroup of disorders of the peripheral nervous system that occur as a result of neuron degeneration.

**Orthostatic hypotension:** low blood pressure when standing up.

**Polygenic risk score (PRS):** a metric of disease risk given by the combined contribution of multiple genetic variants calculated from GWAS statistics.

**Prodromal phase:** a latent time period preceding the clinical diagnosis, in which symptoms appear but clinical diagnostic criteria are not yet met.

**Quantitative trait:** a measurable phenotype that varies between individuals on a continuous scale.

**Regression:** a group of supervised machine learning methods that predict a continuous outcome (real-valued) based on a set of variables.

**Supervised machine learning:** algorithms that learn a relationship between predictors and outcomes based on labelled data.

**Swarm network:** a conglomerate of individual sites with private data (nodes) that exchange model parameters.

**Unsupervised machine learning:** algorithms that identify patterns in unlabelled data.

Precision medicine investigates the plethora of pathophysiologies that are associated with a disorder (Robinson, 2012). Its goal is to offer the best medical care tailored to a patient at a given time. Precision medicine is thus contrasted by the traditional one-size-fits-all approach, whereby a certain treatment is given to all patients suffering from a certain disorder. Oncology was one of the first clinical specialities to adopt this approach (Kupstas et al., 2020; Nowakowski and Czuczman, 2015; Punt et al., 2017), by analysing the genomic landscape of cancer cells to identify cancer subtypes that respond well to certain treatments (Berland et al., 2019; Schmitz et al., 2018; van der Velden et al., 2019). Detailed data are gathered throughout a patient's life, which enables early risk stratification and monitoring of high-risk patients. Prevention and early intervention thus become possible. Furthermore, these detailed data allow the selection of the best available treatment approach for each patient. The prospect of treating PD through a precision medicine approach requires knowledge about the disease mechanisms and treatment targets. Deep phenotyping may aid in the acquisition of such knowledge by guiding clinical trial design and providing insights into disease stratification (Dorsey et al., 2020).

Recently, we have seen the emergence of large, deeply phenotyped cohorts for various disorders, in which valuable clinical, imaging, genetic and biometric data have been collected, often together with longitudinal monitoring, for example the Alzheimer's Disease Neuroimaging Initiative (ADNI) (Jones-Davis and Buckholtz, 2015) and the Parkinson's Progression Markers Initiative (PPMI) (Marek et al., 2018). Such datasets allow researchers to investigate disease aetiologies and the biomarkers of disease progression, and to identify risk factors. In particular, for PD and other complex disorders, with frequent misdiagnoses, unclear disease mechanisms and diverse clinical presentations, deep phenotyping presents the opportunity to fill these gaps in knowledge. Numerous deeply phenotyped PD cohorts are currently available to researchers; nothing comparable has as yet been developed for other genetic disorders.

In this Review, we therefore use PD as a paradigm to introduce deep phenotyping and demonstrate how it can advance precision medicine, in which treatments are tailored to genetically and phenotypically heterogeneous patients. We further discuss recent methodological advances that have allowed us to utilise and understand the large and ever-increasing amount of available data. In particular, we focus on the emergence of deeply phenotyped cohorts in response to advancements in genetic research.

## The imprecise diagnosis and complex genetics of PD

Precision medicine emerged because the traditional one-size-fits-all approach has proven unsuccessful for many disorders. One such disorder is PD, which presents a unique challenge, as its diagnosis remains difficult and its genetic background is diverse. Conventional treatment approaches have thus far been unsuccessful, and a more targeted and personalised approach is required.

### Imprecise diagnosis of PD

PD symptoms result from the progressive loss of dopaminergic neurons in a brain region called the substantia nigra, the primary function of which is motor control. A definitive diagnosis is often challenging, as PD can be confounded with other Parkinsonian syndromes (Williams and Litvan, 2013); however, PD can be distinguished from these by its prolonged response to dopaminergic medication (Williams and Litvan, 2013). Nevertheless, misdiagnosis occurs up to 30% of the time (Schrag et al., 2002). The consequences of these diagnostic errors are dramatic. A recent survey of 2000 people, conducted by the Parkinson's UK charity, revealed that 50% of misdiagnosed individuals with PD receive treatment for a non-existent condition and 6% undergo unnecessary operations or procedures (https://www.parkinsons.org.uk/news/poll-finds-quarter-people-parkinsons-are-wrongly-diagnosed).

In an effort to improve the accuracy of PD diagnoses, the diagnostic criteria for PD in 2015 were updated to include non-motor symptoms (Postuma et al., 2015). The new criteria further include guidance on the use of neuroimaging to rule out PD when no presynaptic dopaminergic deficiency is found. A molecular neuroimaging (Box 1) technique commonly used for this purpose is DaTscan, which involves the injection of a radioactive tracer (Ioflupane, 123-I-FP-CIT) that attaches itself to dopamine transporters on dopaminergic neurons (Djang et al., 2012). DaTscan can discriminate PD from essential tremors and from other non-degenerative tremors (Benamer et al., 2000) and can distinguish PD from healthy controls with high accuracy (Tagare et al., 2017). However, DaTscan cannot differentiate between PD and atypical Parkinsonian disorders, such as multiple system atrophy (MSA) or progressive supranuclear palsy (PSP), which show similar degenerative characteristics. Nevertheless, new magnetic resonance imaging (MRI) techniques, such as neuromelanin-sensitive MRI, and iron-sensitive MRI, are showing promising results that will help to make this distinction and that will further refine PD stratification and prognosis (Prange et al., 2019). Despite their utility for ruling out PD, no neuroimaging techniques are currently recommended for the routine diagnosis of PD. This might be because of the phenotypic heterogeneity of PD, even in brain imaging. For example, a group of patients with a clinical diagnosis of PD but no sign of a dopaminergic deficit in DaTscan has been identified, called scans without evidence of dopaminergic deficit (SWEDD). To date, it is debated whether SWEDD is an early form of PD, a misdiagnosis of clinical PD, or whether it is a distinct movement disorder (Erro et al., 2016; Lee et al., 2021; Marek et al., 2014). At present, the only certain means of diagnosis is the discovery, at autopsy, of depleted brainstem pigmented neurons with Lewy bodies, which are abnormal aggregations of α-synuclein and can be detected histologically. Hence, there is a strong clinical need for the development of accurate, *in vivo* tests at the earliest stages of the disease, for example molecular neuroimaging tracers to visualise α-​synuclein (Shah et al., 2020), and these have recently been successfully developed such that future research in this area will soon clarify the distribution of α-synuclein in the brain (Capotosti et al., 2021)

**Fig. 1.**
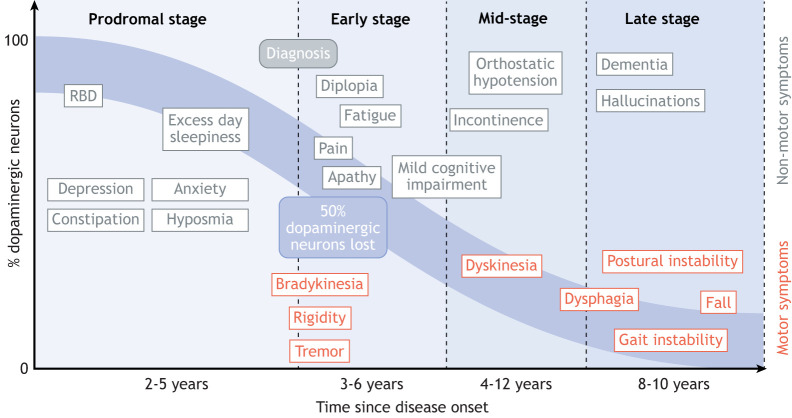
**Parkinson's disease (PD) is characterised by a high degree of heterogeneity.** At diagnosis, >50% of dopaminergic neurons are already lost, and patients can show any combination of motor, neuropsychiatric and autonomic symptoms of differing severity. The blue area indicates the variability in the loss of dopaminergic neurons over time. RBD, rapid eye movement sleep behaviour disorder. See Glossary (Box 1) for descriptions of the symptoms.

One reason why PD is difficult to diagnose is because of the broad variation in its early clinical manifestations (Foltynie et al., 2002) (Fig. 1). Rapid eye movement sleep behaviour disorder (RBD) is a sleep condition characterised by the physical enactment of dreams that are vivid, intense and often violent. RBD often precedes PD, but not systematically before the first clinical motor symptoms (Postuma, 2014). At diagnosis, over 50% of dopaminergic neurons are already lost (Lang and Lozano, 1998), and patients present with a diverse array of neurological, motor and autonomic impairments, each of which also demonstrate variable severity (Foltynie et al., 2002). Disease progression is also highly variable, not only in the rate of decline, but also in the development of additional impairments, such as dementia (Braak et al., 2005; Emre et al., 2007). Clinicians treat dementia with Lewy bodies (DLB) and Parkinson's disease dementia (PDD) as two distinct disease entities. DLB is diagnosed when cognitive impairment precedes Parkinsonian motor signs or begins within 1 year of its onset, whereas PDD develops within the setting of well-established PD (Fig. 5, see ‘1 year rule’) (Jellinger, 2018; Jellinger and Korczyn, 2018). Although this timing distinction is often considered arbitrary, recent neuroimaging and post-mortem studies have demonstrated differences in the quantity and distribution patterns of Lewy bodies and α-synuclein between DLB and PDD, which suggest that these conditions have distinct aetiologies (Jellinger, 2018).

### Imprecise genetics of PD

Most PD cases are currently thought to be sporadic but likely have a genetic component. The heritability of PD is estimated to be ∼26%, with over 90 common variants associated with sporadic PD (Nalls et al., 2019). Individually, these variants have low effects and no clinical utility. However, the overall genetic risk of developing PD can be calculated with polygenic risk scores (PRSs; Box 1). Although people in the highest decile of the PRS distribution are six times more likely to have PD compared to the rest of the population (Nalls et al., 2019), the distributions of PRSs for PD cases and controls are highly overlapping. This means that PRSs for PD currently have a low predictive value for diagnosis and are therefore of limited value for precision medicine.

Around 10% of PD patients have monogenic forms of the disease. The identification of these rare genetic forms was a key step in understanding PD mechanisms. The first identified monogenic cause of PD was a missense mutation within *SNCA*, which causes the p.A53T amino-acid substitution in the α-synuclein protein (Polymeropoulos et al., 1997). This missense is involved in the formation of Lewy bodies, the main hallmark of PD, but is not unique in causing PD. Rare duplications and triplications of *SNCA* (Singleton et al., 2003) also cause PD with a ‘dosage effect’ (Box 1): greater numbers of *SNCA* copies, causing increasing endogenous levels of α-synuclein, have been associated with earlier and more severe clinical symptoms (Eriksen et al., 2005). Moreover, several other PD-causing missense and multiplication mutations have been identified in the *SNCA* gene (Appel-Cresswell et al., 2013; Kruger et al., 1998; Rosborough et al., 2017; Zarranz et al., 2004). To understand the disease mechanisms associated with different genetic variants, we need to capture the full spectrum of variants associated with disease severity and identify the types of symptoms presented by individuals.

**Fig. 2.**
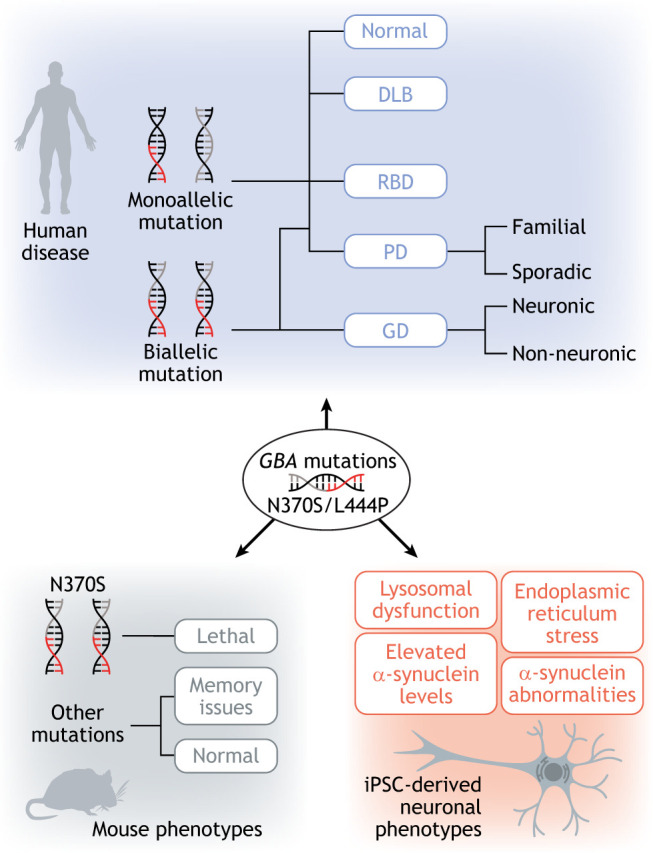
***GBA* mutations: genetic heterogeneity in human versus disease models.** *GBA* mutations are the most common genetic risk factor for Parkinson's disease (PD). However, *GBA* mutations are also found in different human disorders, such as Gaucher's disease (GD), dementia with Lewy bodies (DLB) and rapid eye movement sleep behaviour disorders (RBDs), and in healthy individuals. Different models of *GBA* mutations, including mouse and human induced pluripotent stem cell (iPSC)-derived neuron models, develop the same observable phenotypes. Together, this suggests that other genetic or non-genetic factors contribute to *GBA*-mutant PD in the human population.

The most important and common risk factor for PD is loss-of-function mutations in the glucocerebrosidase gene (*GBA*). These mutations cause lysosomal accumulation of glucocerebroside due to a deficiency in the glucocerebrosidase enzyme, which leads to lysosomal dysfunction (Holleran et al., 1993), resulting in increased levels of α-synuclein via inhibition of the autophagic pathway (Du et al., 2015). However, a key challenge in developing drugs to target *GBA* impairments is that this same mutation can cause multiple disorders, and disease models do not accurately mimic the clinical effects of these mutations in humans (Fig. 2). In Gaucher's disease (GD), a multi-systemic metabolic disorder that typically manifests by adolescence, both homozygous and heterozygous *GBA* mutations increase the risk of developing PD (Riboldi and Di Fonzo, 2019). GD is categorised into three main subtypes with patients exhibiting varied clinical presentations. The most common subtype is non-neuronopathic (Box 1) Type I. The neuronopathic Type II subtype has an earlier onset and is more severe with acute neurological involvement, whereas the neuronopathic Type III subtype has a more chronic presentation (Alaei et al., 2019). There are over 300 pathogenic mutations in *GBA* that affect the structural stability of glucocerebrosidase and reduce its enzymatic activity (Smith et al., 2017). GD Type I patients are frequently associated with N370S *GBA* mutations, while Type II and Type III are typically associated with L444P mutations (Riboldi and Di Fonzo, 2019). However, both heterozygous and homozygous N370S mutations in *GBA* have been found among PD patients with no GD symptoms (Aharon-Peretz et al., 2004). Moreover, an elevated frequency of disease-associated *GBA* alleles has been found among individuals with RBD (Beavan et al., 2015; Gamez-Valero et al., 2018).

The phenotypic heterogeneity induced by these pathogenic variants is uniquely observed in humans. N370S and the L444P are the most frequent *GBA* variants linked to PD (Lesage et al., 2011). In human induced pluripotent stem cell (iPSC) models, these variants consistently cause cellular abnormalities, such as dysfunctional autophagy in the endolysosomal pathway (Fernandes et al., 2016). However, in mouse models, N370S homozygosity is lethal at the neonatal stage of development (Xu et al., 2003), while most other mouse models of *GBA* mutations do not exhibit Parkinsonian phenotypes unless combined with a second risk factor, such as α-synuclein overexpression (Do et al., 2021; Farfel-Becker et al., 2019). This suggests that genetics alone is unable to explain the disease, because other genetic and non-genetic factors, including ageing, oxidative stress and epigenetics, modulate the clinical phenotype associated with *GBA* mutations in the human population.

*LRRK2* encodes leucine-rich repeat kinase 2, also known as dardarin/PARK8*.* G2019S is the most common mutation associated with PD; in some populations, it can be found in 40% of people with this disorder (Lesage et al., 2010). This gain-of-function mutation in *LRRK2* has been associated with a higher risk of PD, and consequently LRRK2 inhibitors have been pursued as a potential avenue for PD treatment (Zhao and Dzamko, 2019). However, whether LRRK2 inhibition in patients is sufficient to reverse or to potentially prevent PD manifestation is currently debated. One reason for this scepticism is the incomplete genetic penetrance of the G2019S mutation, which suggests that other risk factors alter disease risk in carriers of this variant. Another reason is that some G2019S carriers exhibit the clinical manifestations of PD without developing Lewy bodies (Kalia et al., 2015).

Although PD patients with *LRRK2* and *GBA* mutations represent a small fraction of PD cases, they are nevertheless crucial for precision medicine. This is because by understanding the genetic and phenotypic heterogeneity of these mutations we can hopefully develop successful therapies to reverse their effects in PD patients. As sporadic patients represent the majority of PD cases, genomic data might also play an important role in extending the application of these therapies to sporadic patients with *GBA*/*LRRK2*-associated mutations, should these mutations be identified in this cohort. Indeed, some GBA mutations have already been reported to exacerbate disease outcome in sporadic patients and were associated with accelerated development of dementia and a more aggressive motor course (Stoker et al., 2020).

This illustrates that improving our understanding of genetic heterogeneity and how it corresponds to clinical variability should increase our ability to both predict disease and define subtypes by their aetiology, thus paving the way for more precise treatments (Hennekam and Biesecker, 2012). To achieve the aim of providing patients with tailored treatment that considers their unique genetic and phenotypic presentation, a deep understanding of the phenotypes and genetics of a disorder is needed. Precision medicine hence requires such fine-grained, deep data.

## Precision medicine requires deep genomic and phenotypic data

As exemplarily presented for PD, a clinical disorder can be associated with diverse clinical phenotypes that render diagnosis difficult and with a diverse genetic background that renders treatment development and selection difficult. The one-size-fits-all approach does not account for such heterogeneity. Precision medicine could provide more tailored treatments, but achieving this requires deep understanding of the disorder, which necessitates in-depth data collection and analysis.

### Why do we need better phenotypes?

The genetic and phenotypic heterogeneity observed within complex disorders complicates research. If a heterogeneous patient group is described by a single label, as often occurs in case-control studies, any subsequent analysis of this group will inherit the uncertainty and confounders from this broad diagnostic label. This has a negative impact on clinical practice, which relies on insights gained from such studies. To improve research outcomes, broad clinical labels should be replaced by sensitive, objective and detailed phenotypes.

**Fig. 3.**
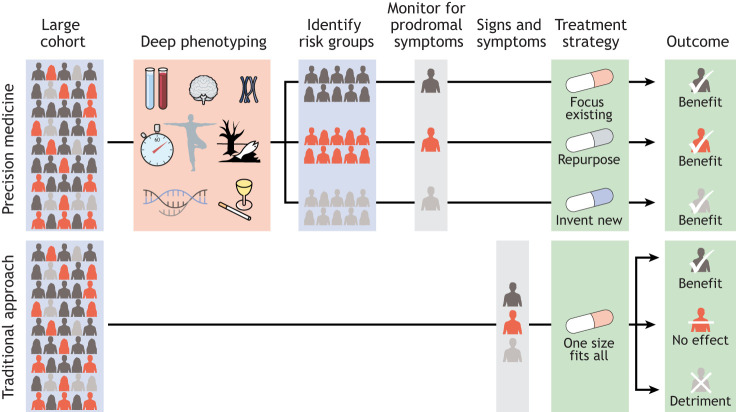
**The merit of deep phenotyping for precision medicine.** In traditional clinical practice, the same treatment strategy is applied to anyone diagnosed with a disorder. This means that a diagnosis is made and a treatment given based on a predefined set of signs and symptoms. The outcome, including treatment effectiveness, can thus be varied due to heterogeneity within that disorder. Precision medicine uses fine-grained information gained through deep phenotyping and genetics to match the best treatment to an individual patient. In addition, it can aid in the monitoring and identification of at-risk individuals and enable preventative interventions.

Medical intervention research relies heavily on clinical trials, in which the effectiveness of a treatment is compared between groups. Given that the aforementioned broad diagnostic labels can capture multiple aetiologies, we may well see heterogeneous responses to treatment in a clinical trial, with only a small subset of patients showing a benefit (Fig. 3). The overall verdict in such cases would be that the treatment is not effective, despite its efficacy on a particular subset. This one-size-fits-all approach may partly explain why many clinical trials investigating disease-modifying drugs for PD have failed (Athauda and Foltynie, 2016). As such, clinical trials could greatly benefit from more granular stratifications of PD and from more personalised approaches.

When several treatments for a disorder successfully pass clinical trials, treatment selection becomes a difficult task for clinicians. Clinicians mostly rely on clinical expertise and general treatment guidelines when deciding which treatment to prescribe, creating a long journey of trial-and-error until a suitable treatment is found. Treatment selection could thus benefit from the insights of subtype-sensitive clinical trials and a general investigation into the association of treatment effect and biotypes. For example, clinical trials assessing treatments that target genetic forms of PD are starting to become more common (Mullin et al., 2020; Schneider and Alcalay, 2020), despite these forms being much rarer than sporadic PD. As precision medicine aims to consider the pathophysiological uniqueness of an individual as well as the genetic background, understanding the genetic basis of a disorder is an important first step in this direction.

Phenotypes that are accurate, sensitive and robust are important, as the quality of the measurement determines the utility of the analysis, especially so for genetic studies (O'Sullivan and Ioannidis, 2021). In a genetic association analysis, if the case group contains control and/or misdiagnosed subjects, disease-associated genetic loci may not be identified (Manchia et al., 2013). In addition, the selection of controls must be monitored closely as some controls, despite being healthy at study onset, may go on to develop a disorder at a later stage. In such a situation, we need better defined and more precise phenotypes to identify genetic associations rather than solely prioritising larger sample sizes for better statistical power (Manchia et al., 2013). Pastor (2012) identified two objectives for improving phenotype quality and hence the quality of genetic analysis: (1) the confirmation of clinical diagnoses through long-term follow up or additional biomarker tests and scans; and (2) the differentiation of sub-phenotypes or the usage of traits that more accurately reflect the disease spectrum. Quantitative traits (Box 1) have been shown to have better reproducibility in GWAS compared to binary traits, which often encompass broad diagnostic classes with inherent heterogeneity (O'Sullivan and Ioannidis, 2021). For example, the genetic associations for height measured in centimetres are more likely to be reproducible across different cohorts compared to genetic associations for the binary trait of being taller than 180 cm. Recent efforts to investigate the genetic basis of more precise phenotypes include studies that reveal the heritability of image-derived phenotypes (IDPs), like brain region volumes or cortical thickness measurements (Elliott et al., 2018). Thus, unbiased, objective and sensitive measures are needed to describe phenotypes.

With the emergence of next-generation sequencing, Hennekam and Biesecker (2012) foresaw the need for next-generation phenotyping back in 2012. A decade later, deeply phenotyped cohorts have become a major subject of interest for clinicians and medical geneticists, as we discuss next.

### What is deep phenotyping?

**Fig. 4.**
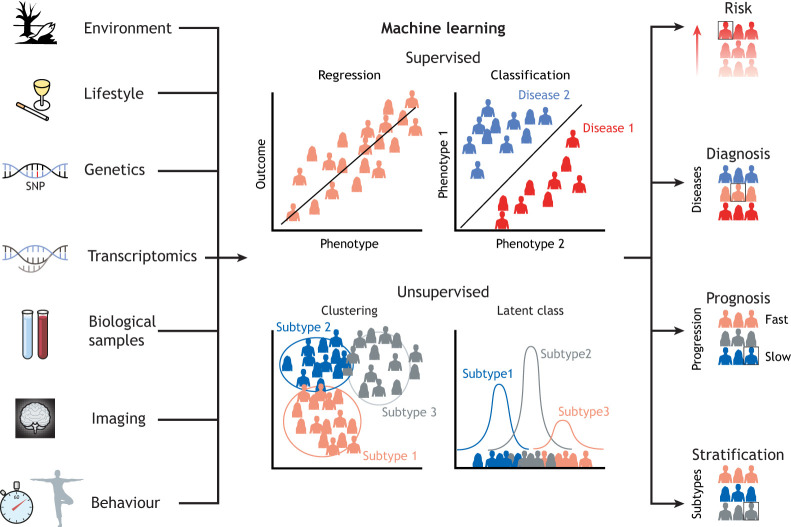
**Towards precision medicine by integrating multi-modal biomedical data.** A number of the research objectives can be explored with genetic data and deep phenotyping. Deep phenotyping provides data on many different scales, such as environmental factors, lifestyle, multi-omics, diverse biological samples, imaging, behaviour, etc. Such complex data benefit from the advent of machine learning, such that large-scale, heterogeneous, multi-modal phenotypic and genetic data can be translated into meaningful information about risk, diagnosis, prognosis and stratification.

In clinical practice, a phenotype is a label assigned to a specific set of observable traits, including, among others, morphological, physiological and/or behavioural traits (Robinson, 2012). Such traits can be inferred from medical history, questionnaires, clinical tests, blood tests, imaging and/or physical examinations (Fig. 4). Instead of reducing this highly complex set of traits into one disease label, deep phenotyping aims to retain this information. It tries to capture an individual's phenotypic presentation in a precise and comprehensive manner by leveraging information gained from different data sources (Robinson, 2012). These metrics are also monitored over time, instead of focusing on a single time point when a diagnosis is made (Weng et al., 2020). As a result, an individual's specific phenotype is described in all of its dimensions. Deep phenotyping thus offers a more complete picture of a disorder so that its nature, treatment and subtypes can be better understood (Dorsey et al., 2020) (Fig. 3).

Deep phenotyping also provides measures at different scales, such that the journey of a given protein can be followed from the level of genetics through omics and all the way through to manifestations in behaviour. A phenotypic assessment on different scales thus gives a better understanding of disease manifestations, their impact on daily life and their relation to pathophysiology. Combined with genetic data, which are becoming increasingly accessible due to falling costs, we can explore the heritability and true genetic basis of objective and precise phenotypes (O'Sullivan and Ioannidis, 2021). Such deep understanding can guide drug discovery and advance precision medicine in an objective and effective manner.

### How can we analyse and utilise deep phenotyping data?

Traditional research primarily relies on hypothesis-driven approaches, using which specific data are gathered to answer one question. Data-driven approaches have gained popularity following advances in data collection, storage and computing. Deep phenotyping produces an abundance of high-dimensional data that can be leveraged to answer a multitude of questions. Such an abundance of data also allows for data-driven approaches (Goecks et al., 2020).

The abundant data generated by deep phenotyping, however, pose unique challenges to statistical methods. Deeply phenotyped cohorts offer a large amount of data for a comparably small number of individuals. Despite global efforts, including the sharing of data, to obtain a more representative number of participants, cohorts remain small in relation to the number of collected features; for example, the PPMI study collected 2442 measures on 1683 individuals. Therefore, special care must be taken when performing statistical tests and building models. Additionally, large amounts of missing, highly correlated or multi-modal data pose further challenges to conventional statistics in these cohorts. This is because standard statistical tools often fail to account for such data characteristics (Johnstone and Titterington, 2009).

Machine learning (ML) approaches are, therefore, required to handle such challenging data (Goecks et al., 2020). There are four broad applications of ML that are relevant for medicine: risk analysis, diagnosis, stratification and prognosis (Fig. 4). A major aim of precision medicine is to identify people at risk early, such that preventive measures can be applied. Risk analysis, as well as diagnosis, can be achieved through supervised ML (Box 1) techniques like regression (Box 1) and classification (Box 1) methods that reveal potential risk and protective factors (Solana-Lavalle and Rosas-Romero, 2021). Another aim of precision medicine is to provide tailored treatment to individual patients, which can be achieved by classifying patients into finely grained disease subtypes that respond better to certain treatments. Unsupervised ML (Box 1) methods like clustering (Box 1) approaches and latent class (Box 1) can reveal such subtypes (Brendel et al., 2021). Precision medicine also aims to identify treatment tailored to a specific stage of a disorder. Knowledge about these different aspects can be gained through disease progression modelling (Oxtoby et al., 2021).

Data generated through deep phenotyping therefore pose challenges; however, powerful methods, mainly from the field of ML, exist and are being developed to handle them. When such methods are successfully applied to rich and valuable datasets, we can answer important questions about disorders and thus advance precision medicine.

## Data collected for deep phenotyping and the insights gained

Precision medicine requires deep phenotyping, and methods exist to handle and analyse such data to provide useful insights into disorders. Here, we discuss how such valuable data can be collected and what information can be gained through each modality.

### Deep clinical phenotyping

Clinical phenotyping in PD often uses information from clinical tests, questionnaires or subjective descriptions of an individual's tremor to assign a disease label. Deep clinical phenotyping begins with a traditional clinical examination, in which such data are gathered, and then expands on this information with sensors that monitor patients over longer periods in real-life situations (Dorsey et al., 2020).

Traditional clinical examinations already provide information about phenotypic heterogeneity that can be leveraged to study subtypes. Collected data can include clinical tests and examinations for motor impairment, autonomic function and cognitive abilities, as well as questionnaires about mental health, sleep quality and problems with the activities of daily living. The identification of PD subgroups has been a research focus since 1990 (Jankovic et al., 1990). Instead of studying differences between cases and controls, differences between cases can be studied through data gathered in clinical examinations that are then analysed with ML methods. Early efforts focus on motor symptoms of PD assessed with the Unified Parkinson Disease Rating Scale (UPDRS) and differentiate three subtypes based on the ratio of the summed scores of specific domains: tremor dominant, postural instability and gait difficulty, or akinetic rigid and intermediate (Kang et al., 2005; Schiess et al., 2000). The inclusion of non-motor symptoms increases the stability and consistency of these subtypes (Ren et al., 2021). Efforts to include a broader range of clinical examinations and apply clustering techniques have identified discrete PD clinical subgroups, each displaying a characteristic set and degree of symptoms (Fereshtehnejad et al., 2015; Lawton et al., 2015). The subtypes revealed by Fereshtehnejad et al. (2015) have been subsequently shown to predict disease progression (De Pablo-Fernandez et al., 2019). As Fereshtehnejad et al. (2017) noted, several studies reveal clinical subtypes identified through ML approaches, but no consensus has been established, nor have these methods been incorporated into clinical practice. This might be because the proposed subtypes and the methods used to define them lack consistency and stability (von Coelln et al., 2021). The inconsistency in such methods could be due to the selection of different variables for the model and the instability could be due to a selection bias introduced via data cleaning (Fereshtehnejad et al., 2017). Furthermore, the longitudinal aspects of PD have thus far been disregarded in most of these approaches, such that snapshots of patients at different stages of the disorder have been used. Owing to these issues, more fine-grained and consistently collected data that better represent the clinical phenotype or incorporation of other phenotype modalities and the application of methods that combine clustering and progression modelling (Young et al., 2018) could improve stratification efforts.

Data-driven PD diagnosis does not focus on traditional clinical examinations; instead, it uses these as the prediction target. As PD is a clinical diagnosis, a diagnosis based on clinical examinations is straightforward and does not require ML. However, a recent study showed that data gathered through other means, e.g. voice recordings, gait analysis, etc., have resulted in good prediction of PD using ML methods (Mei et al., 2021).

#### Digital sensors

Clinical phenotypes gathered through traditional clinical examinations have several limitations. First, detailed clinical tests and questionnaires have been criticised for their lack of precision (Regnault et al., 2019). Second, clinical tests are conducted at specific time points and only reveal snapshots of a person's phenotype. Such snapshots can be confounded by the increased phenotypic variability observed with ageing and with disease onset and progression (Sheridan et al., 2003). Third, detailed investigations by clinicians are time consuming and expensive. These specifically designed tests can take several hours and are conducted by trained staff, meaning that participants must attend clinics or be assessed at home. If an impairment is too advanced, patients may drop out due to the time investment and strain of the procedures (Dorsey et al., 2020). Finally, clinical tests are conducted in an artificial environment and do not accurately reflect real-world circumstances (Dorsey et al., 2020).

Digital sensors, which collect data and convert and transmit them digitally, can address these limitations (Brognara et al., 2019). Such devices tend to be more sensitive and accurate than traditional approaches. They also enable long-term data collection, which can provide a clearer picture of phenotypes and their trends by averaging measures over longer periods of observation (Hayes et al., 2008). In addition, these digital sensors allow automatic, non-disruptive data collection, in a real-world setting that does not depend on experienced staff. For example, in addition to extensive biannual assessments, the Personalized Parkinson Project (PPP) collects day-to-day real-world data through a wearable smart device known as the Verily Study Watch (Bloem et al., 2019). Participants are asked to wear the device all day throughout the 2 years of the study. This multi-sensor device collects data about acceleration, pulse rate, electrodermal activity, electrocardiogram, relative humidity, environmental temperature and ambient light level. Preliminary analyses show that such digital data have promising features that discriminate healthy controls from PD patients and that sensitively describe motor symptom progression (Schlachetzki et al., 2017; Shah et al., 2020).

Digital sensors provide large amounts of data and thus power for statistical analyses: a considerable number of observations are acquired per person per second over a long time. Such sensors can also be worn by anyone and are relatively inexpensive and non-invasive. For comparison, polysomnography monitors the sleep of a single person over a single night in an artificial sleep laboratory, which is both costly and an inconvenience for the participant. By contrast, wrist-worn accelerometers can provide data about sleep for many participants over several nights at home (Sundararajan et al., 2021). Although the sleep features assessed by wearable sensors do not match polysomnographies perfectly, they provide valuable and valid information about numerous clinical features about sleep, steps taken, physical activity, distance, etc. for many people (Evenson et al., 2015), and thus help us gain longitudinal insights into impairments in everyday life (Johansson et al., 2018).

### Biomarkers and intermediate phenotypes

One strength of deep phenotyping is that it captures phenotypes at different scales and enables the study of biomarkers, which are endogenous, measurable, characteristics that mark either the risk for, or the manifestation of, a disease. Biomarkers allow deeper understanding of ongoing changes in disease pathology, from the molecular to the behavioural level. For example, changes in the brain can be detected via medical imaging, while cellular perturbations can be detected through omics measures.

**Fig. 5.**
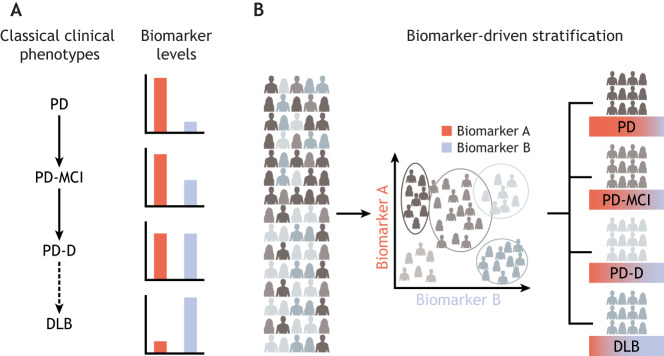
**Clinical phenotype-driven versus biomarker-driven research.** (A) Biomarkers are useful for identifying differences between clinical phenotypes and clinical subgroups, and in providing a differential diagnosis. (B) Biomarkers can also differentiate disease subtypes, which can then be associated with clinical phenotypes and behaviour. For example, in patients with pure synucleinopathy, we expect to only see PD-specific biomarkers (red), whereas in those with AD co-pathologies, we expect abnormalities in AD-specific biomarkers as well (blue). The distinction between DLB and PDD is defined by the '1-year rule': if the onset of dementia symptoms is within 1 year of parkinsonism, the disorder is called DLB; if parkinsonism is present for more than 1 year before the onset of dementia, the disorder is called PDD. AD, Alzheimer's disease; DLB, dementia with Lewy bodies; MCI, mild cognitive impairment; PD, Parkinson's disease; PD-D, Parkinson's disease dementia.

These quantitative traits can be used to study the differences between clinically defined groups. However, like in genetic analyses, the inherent uncertainty and imprecision of binary disease labels affect such studies, especially in neurodegenerative disease research (Mattsson-Carlgren et al., 2020). An alternative approach is to objectively identify homogeneous groups based on biomarkers and then explore the association between these groups and clinical phenotypes (Espay et al., 2017) (Fig. 5). Methods like Mendelian randomisation (Box 1) can help identify causal links between genes and environmental factors or biomarkers (Noyce and Nalls, 2016). Thus far, a limited number of biomarkers have shed light on the neuropathophysiology of disease subtypes and have been helpful for monitoring disease progression and predicting its course. For example, the cerebrospinal fluid (CSF) biomarkers amyloid-β (Aβ42), total tau and phosphorylated tau can serve as early markers of Alzheimer's disease and thus provide clinically relevant diagnostic information (Blennow and Zetterberg, 2018). Other biomarker modalities that assess molecular markers, like CSF and blood, or positron-emission tomography (PET) have shown great prospects in understanding disease mechanisms and spreading of pathologies in neurodegeneration (Lashley et al., 2018).

#### Blood and CSF biomarkers

The Alzheimer's disease examples highlighted above (Blennow and Zetterberg, 2018; Lashley et al., 2018) show that blood and CSF can be useful sources of biomarkers for neurodegenerative disorders. To aid prognostic and diagnostic decision-making, biochemical markers of early PD have also been extensively studied. However, no single marker has so far been sufficient to accurately diagnose PD. For example, astrocytic cell death in PD can be detected by elevated blood and CSF levels of glial fibrillary acidic protein (Ding et al., 2021). However, this signature is also observed in MSA, PSP and corticobasal degeneration, thus complicating the differentiation of typical and atypical Parkinsonian disorders (Constantinescu et al., 2010). Conversely, neurofilament light protein levels can distinguish PD from PSP and MSA, but are unable to distinguish between PSP and MSA (Constantinescu et al., 2010). As such, a repertoire of biomarkers needs to be studied simultaneously to aid the diagnosis of PD (Schapira, 2013).

The loss of dopaminergic neurons in PD likely involves inflammation, either as a cause or a consequence (Appel, 2012). A recent study reported the appearance of an α-synuclein-reactive T-cell population in the blood 10 years prior to diagnosis with motor PD (Lindestam Arlehamn et al., 2020). This result suggests that it might be possible to identify modifications in the blood of individuals prior to developing symptomatic PD or another α-synucleinopathy, such as DLB and MSA. As such, changes in the blood's transcriptome, as obtained by RNA profiling, could identify novel PD biomarkers.

#### Brain imaging biomarkers

Brain imaging offers rich, detailed *in vivo* data that can assist with differential diagnosis, prognosis and subtyping (Pagano et al., 2016). Various imaging modalities exist that can investigate structural, functional and molecular changes in diseased brains.

Structural MRI with T1 weighting is the most commonly available standard brain imaging resource in deeply phenotyped cohorts. Structural imaging measures from such cohorts have revealed neuroanatomical PD subgroups that correspond to clinical subtypes and that can predict disease progression (Shu et al., 2021; Wang et al., 2020). Some cohorts offer molecular imaging data that can be used to research the spreading patterns of proteins in the brain. Some cohorts (Table 1) include DaTscan imaging, which can shed light on the SWEDD subgroup (Choi et al., 2017). In general, molecular imaging has been used to investigate the spreading pattern of α-synuclein in PD (Horsager et al., 2020) and to identify distinct subtypes. Such insights are valuable for precision medicine as the identification of biotypes, which are clusters of individuals that share biological signatures, can inform treatment responses in several disorders, such as cancer and Alzheimer's disease (Cattaneo et al., 2016; Machado et al., 2020).

**Table 1.**
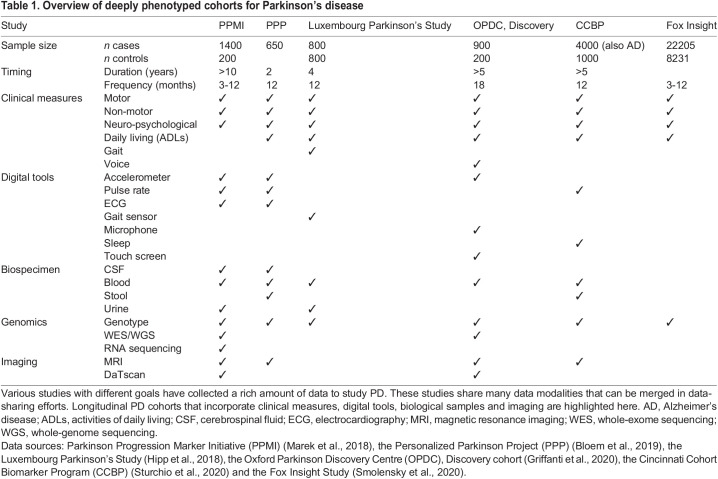
Overview of deeply phenotyped cohorts for Parkinson's disease

Imaging data from deeply phenotyped cohorts are becoming increasingly available to non-imaging experts in the form of IDPs, which provide a great tool for studying the brain. IDPs summarise high-dimensional data as informative, subject-level measures of thickness, volume, connectivity or protein levels. These measures can either be curated from expert knowledge or acquired in a data-driven objective manner (Gong et al., 2021). IDPs can be linked to genetics to reveal the genetic contributions to brain abnormalities relevant for psychiatric and neurological disorders, as well as for ageing. For example, a GWAS with IDPs using data from the UK Biobank has shown that many brain characteristics are heritable and some genes, such as *EGF*, are associated with brain lesions (Elliott et al., 2018).

### Cohorts and data sharing

Several PD cohorts now exist that provide data from diverse sources, enabling research into the complexity of the disease. Such cohorts include the aforementioned PPMI (Marek et al., 2018), the Oxford Parkinson Discovery Centre Discovery cohort (Griffanti et al., 2020), the PPP (Bloem et al., 2019), the Cincinnati Cohort Biomarker Program (Sturchio et al., 2020), the Luxembourg Parkinson's Study (Hipp et al., 2018) and the Fox Insight Study (Smolensky et al., 2020) (Table 1). Although these cohorts follow different objectives, they share a vast amount of common data modalities that could be merged to increase the sample size, e.g. for GWAS. Data-sharing efforts are needed to create larger, more unbiased population samples that better capture heterogeneity, especially in terms of genetics. Platforms like the Dementia Platforms UK (Koychev et al., 2020) are set up to combine data from several cohorts into a standardised framework. A similar tool for PD is still required, despite several efforts and calls for it, for example by the BioLoC-PD working group (Heinzel et al., 2017). However, some efforts to combine and harmonise PD cohorts do exist, such as the Accelerating Medicines Partnership Parkinson's Disease platform (Iwaki et al., 2021).

As medical data are sensitive and require protection, indirect ways in which to securely share such data are being explored. Instead of defining data-sharing agreements between study sites, decentralised approaches, such as swarm learning (SL), can be followed. SL does not require data exchange or a central structure. Instead, parameters are trained by local models on local data and are included in a swarm network (Box 1) that consists of multiple local sites (Warnat-Herresthal et al., 2021).

In addition to the technical and legal challenges of data sharing, data storage, analysis and transferability issues are also a concern. These are discussed in the following section.

## Challenges and opportunities

**Fig. 6.**
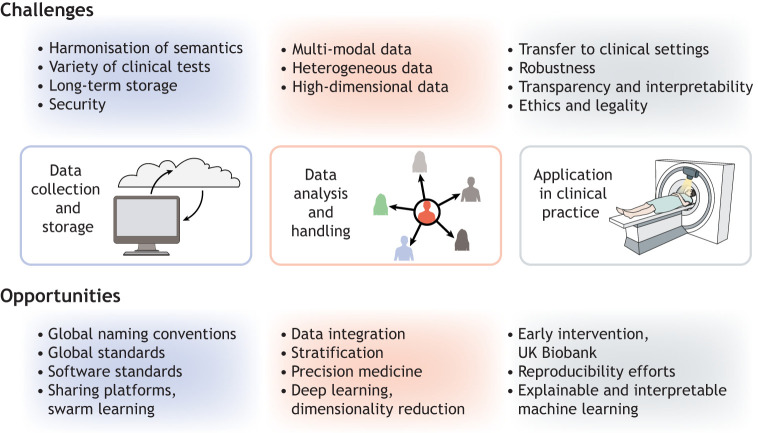
**Overview of challenges and resulting opportunities.** Deep phenotyping produces large amounts of data, which present various challenges in three domains: data storage, analysis and application. However, these challenges give us the opportunity to set global standards and, once the infrastructure is in place, to gain valuable novel insights into disorders that can guide us to precision medicine.

New techniques may enable the faster and easier collection of large amounts of data, but they also pose new challenges in the curation, integration, sharing and interpretation of the data (Fig. 6). Data collection and storage require agreed-upon standards and global efforts. The analysis of these data is complicated by the sheer heterogeneity, multi-modality and scale of the data. Furthermore, one of the biggest challenges lies in transforming these complex data into medically actionable resources with clinical utility. Here, we focus on the data analysis aspect, as the other elements have recently been reviewed elsewhere (Matrana and Campbell, 2020; Weng et al., 2020).

### How to merge data modalities?

Deep phenotyping produces different data modalities that have to be studied together. The classical method used to integrate different data sources is to merge different modalities into a single matrix. However, this can introduce a bias, as higher-dimensional data (e.g. imaging), unlike lower-dimensionality data (e.g. demographics), will often be preferred by algorithms simply because of their size and thus larger influence. Markello et al. (2021) have proposed creating a similarity network for each data modality separately and then iteratively fusing these together. This similarity network fusion approach has the advantage of overcoming the dimensionality bias, plus it generates a low-dimensional representation of each source that can be interpreted.

Another issue is the noise associated with each modality. Gene network methods are widely used to identify perturbed molecular pathways that underlie complex genetic disorders. A common limitation of all these approaches stems from the functional datasets themselves, as no single dataset or data modality can provide a complete picture of the functional association between genes. Although it is possible to merge different datasets and to build a more comprehensive and unique network, different levels of noise from each data source get incorporated into the result, which can lead to false associations. Honti et al. proposed a powerful method to address this issue by weighting functional similarities between genes according to their likelihood of influencing the same mammalian phenotype(s) (Honti et al., 2014; Sandor et al., 2017).

#### Analysing imaging and genetic data

New fields of study have emerged that deal with the combination of different data modalities. Imaging genetics integrates analysis of brain imaging and genomics to gain insights into the genetic impact on brain function and structure. In early efforts, a single genetic marker (e.g. a single-nucleotide polymorphism) and a single imaging trait were studied, and then PRSs and multiple IDPs were studied together (Shen and Thompson, 2020). Today, methods that integrate multiple single-nucleotide polymorphisms and multiple traits exist that model the influence of genetic variation on several IDPs. To decrease the amount of parameters needed to fit such univariate models, sparse multivariate models have emerged in which all features are integrated into a single large model. This further allows us to model the relationship between genetics and phenotypes while accounting for dependencies between phenotypes (Nathoo et al., 2019).

#### Analysing imaging and transcriptomic data

Analogously to imaging genetics, imaging transcriptomics deals with the integrated analysis of brain imaging and gene expression data to gain insights into the molecular changes associated with neurodegeneration. As omics offer a dynamic dimension, disease progression can be followed by using imaging transcriptomics (Katrib et al., 2016). A common approach is to correlate gene expression with IDPs through shared defined regions of interest (Mroczek et al., 2021). This means that a discrete map is applied to the brain in which all measures in one region are summarised to represent that region. This is largely made possible through the publicly available dataset from the Allen Human Brain Atlas (https://human.brain-map.org/), which holds gene expression data for 102 brain regions and for 20,000 genes of six post-mortem brains from healthy donors (Hawrylycz et al., 2012). Guidelines to handle this dataset have been proposed to standardise the research in this emerging field (Arnatkeviciute et al., 2019). Recent efforts have attempted to utilise the spatial resolution of both data sources (Zarkali et al., 2021).

### Heterogeneity as an opportunity

In disease research, the problems arising from high heterogeneity should also be viewed as an opportunity. Past efforts to investigate PD have led neither to a successful understanding of the disorder nor to a disease-modifying treatment. Thus embracing data heterogeneity and investigating it could provide new insights. Several methods for uncovering heterogeneity in large datasets have been proposed. We can classify these approaches as subtype and stage models: subtype models focus on finding homogeneous subgroups while ignoring the disease stage; stage models ignore subtype heterogeneity but investigate the disease stage. The SuStaIn model (Young et al., 2018) combines both of these efforts by integrating clustering and disease progression modelling. It has successfully shed light on Alzheimer's disease subtypes based on the spreading of phosphorylated tau (Vogel et al., 2021) and could inform spreading patterns and progression subgroups in PD as well.

### How to handle large data?

Deep phenotyping combined with genetic data has led to the generation of unprecedented amounts of data, which comes with its own set of challenges. First, the storage and handling of high-dimensional data is very computationally demanding. This issue is typically addressed through the use of high-performance computing systems, by sharing resources among research institutes and by using cloud-based systems (Bauermeister et al., 2020). Second, the analysis of high-dimensional data requires large sample sizes to provide sufficient statistical power. Data-sharing efforts and the decreasing costs of data collection are helping in this regard (Iwaki et al., 2021). Third, appropriate methods to process such data need to be developed and applied. Typically, dimensionality reduction (Box 1) techniques are used to extract meaningful features that can be interpreted (Tao et al., 2017). An alternative approach is deep learning (DL; Box 1). Despite its debated role in medicine due to a lack of transparency and model interpretation, DL is gaining popularity for its ability to handle high-dimensional datasets. Especially in medical imaging, convolutional neural networks (CNNs; Box 1) are often applied with good results (Choi et al., 2017). The concerns regarding transparency are being addressed through the branch of interpretable and explainable artificial intelligence that, for example, generates visualisations of the decision process, such that physicians can review the decision made by the model (Magesh et al., 2020).

### From deeply phenotyped cohorts to the general population

**Fig. 7.**
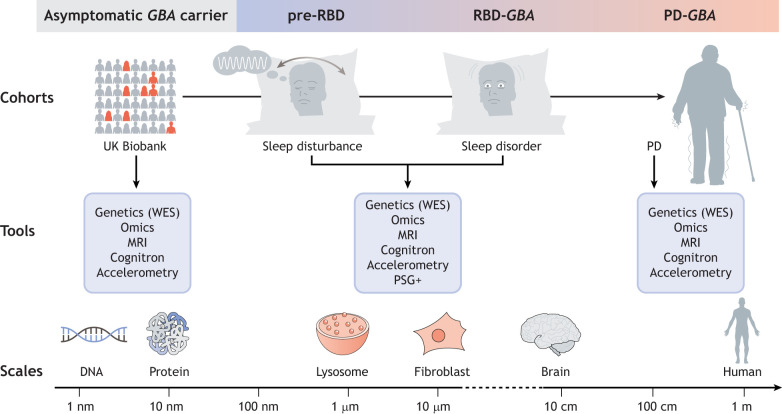
**A model for precision medicine in diagnosing and treating PD.** To evaluate how pharmacological interventions might reverse the early (pre)clinical symptoms of PD, the features of *in vitro* disease models, such as lysosomal dysfunction in fibroblasts with *GBA* mutations, need to be linked to the phenotypes of RBD/PD patients, such as their sleep and biomarker profiles. The UK Biobank population could also be profiled to identify the earliest features of RBD and thus help more at-risk patients. Cognitron is an artificial intelligence tool to evaluate mental skills of an individual (Hampshire et al., 2021; https://www.cognitron.co.uk/). *GBA*, glucocerebrosidase gene; MRI, magnetic resonance imaging; PD, Parkinson's disease; PSG, polysomnography; RBD, rapid eye movement sleep behaviour disorder; WES, whole-exome sequencing.

Although deeply phenotyped cohorts constitute a unique opportunity for precision medicine, the collection and analysis of certain data modalities are time consuming for clinicians, patients and researchers alike. This means that participation cannot be extended to the general population nor to cohorts that include hundreds of thousands of individuals. One such resource-heavy data modality is the definitive diagnosis of RBD using polysomnography, which monitors various body functions during sleep in a specialised clinic (Hogl and Stefani, 2017). Identifying RBD in the general population is crucial as RBD patients that carry severe *GBA* variants show faster transition to PD and dementia (Krohn et al., 2020). Fortunately, *in vitro* models have also highlighted a possible alternative diagnostic tool for RBD based on findings that implicate lysosomal storage dysfunction as an early marker of GBA deficiency (Bae et al., 2015). These insights, combined with data resources such as the UK Biobank, offer a unique opportunity to identify severe *GBA* variant carriers or individuals with sleep disorders. The UK Biobank provides a broad range of phenotypic data, including cognitive and sleep measures, digital markers like movement recorded by smartwatch accelerometers, genetic information and, more recently, blood proteomic profiles for over 500,000 adults aged 37-73 years (Bycroft et al., 2018). To expand the search for individuals in the prodromal phase of RBD in the general population, we need to assemble such diverse sources of data across different scales, spanning *in vitro* cellular models and clinical cohorts, as well as the general population (Fig. 7). Additionally, using novel computational approaches, features of *in vitro* cellular models have to be linked to biomarker profiles of deeply phenotyped patients on an individual level and expanded to the UK Biobank population to identify the earliest features of disease.

Precision medicine has been criticised for its lack of clinical utility and its failure to address the demands of public health (Ramaswami et al., 2018). With key exceptions, such as the UK Biobank, which combines principles of deep phenotyping with general health aims (Bycroft et al., 2018), most deeply phenotyped cohorts focus on one disorder and explore it in depth with a strong emphasis on clinical interventions and drug research. On the surface, this focus on one disorder can be regarded as a financial investment without much benefit for the general population. However, methods developed for deeply phenotyped cohorts, and the research insights into disease mechanisms and risks, provide valuable information for the general public. Furthermore, identifying disorders earlier could achieve a shift from treatment to prevention, which would greatly benefit the general population.

#### Future perspectives

Deeply phenotyped cohorts offer tremendous opportunities to advance our understanding of complex disorders. The observed phenotypic and genetic heterogeneity of such diseases must be addressed to understand their underlying mechanisms and to provide targeted treatments. High-throughput sequencing technologies provide insights into genetic heterogeneity, while deep phenotyping provides insights into phenotypic heterogeneity via clinical (and intermediate biological) phenotypes. These complex data challenge traditional statistical methods, but advances in ML and data-sharing efforts show how such data can be translated into meaningful and clinically valuable information.

One of the biggest challenges is to transfer disease insight captured in deeply phenotyped cohorts to the general population and dissect the prodromal phase of a disorder. Gathering as much in-depth data from the general population is not feasible, but it is done for deeply phenotyping cohorts. Therefore, novel approaches to transfer our insights to clinical practice are needed. As most cohorts focus on specific disorders, they provide limited merit to the general population. Nevertheless, such shortcomings can be addressed by the wealth of data provided by public resources, such as the UK Biobank, that pose a unique opportunity to align clinical cohorts to the general population. This would require diverse skills and expertise in diverse areas, including clinical, cellular, genomic, pharmacological, computational and artificial intelligence, to come together and embark on interdisciplinary collaboration to push the boundaries of scientific research. Therefore, through combined efforts of industry and academia, the goal of precision medicine is reachable for PD and other complex disorders.
